# Experimental Infections of Pigs with African Swine Fever Virus (Genotype II); Studies in Young Animals and Pregnant Sows

**DOI:** 10.3390/v14071387

**Published:** 2022-06-25

**Authors:** Louise Lohse, Jens Nielsen, Åse Uttenthal, Ann Sofie Olesen, Bertel Strandbygaard, Thomas Bruun Rasmussen, Graham J. Belsham, Anette Bøtner

**Affiliations:** 1 National Veterinary Institute, Technical University of Denmark, Lindholm, DK-4771 Kalvehave, Denmark; lolo@ssi.dk (L.L.); jeni@aqua.dtu.dk (J.N.); asut@vet.dtu.dk (Å.U.); bstr@ssi.dk (B.S.); tbru@ssi.dk (T.B.R.); grbe@sund.ku.dk (G.J.B.); 2 Statens Serum Institute, Artillerivej 5, DK-2300 Copenhagen S, Denmark; asjo@ssi.dk; 3 National Institute of Aquatic Resources, Technical University of Denmark, DK-2800 Kongens Lyngby, Denmark; 4 Department of Veterinary and Animal Sciences, University of Copenhagen, Grønnegårdsvej 15, DK-1870 Frederiksberg, Denmark

**Keywords:** African swine fever, domestic pigs, experimental infection, virus transmission

## Abstract

African swine fever is an important viral disease of wild and domestic pigs. To gain further knowledge of the properties of the currently circulating African swine fever virus (ASFV), experimental infections of young pigs (approximately 8 weeks of age) and pregnant sows (infected at about 100 days of gestation) with the genotype II ASFV Georgia/2007 were performed. The inoculated young pigs developed typical clinical signs of the disease and the infection was transmitted (usually within 3–4 days) to all of the “in contact” animals that shared the same pen. Furthermore, typical pathogical lesions for ASFV infection were found at necropsy. Inoculation of pregnant sows with the same virus also produced rapid onset of disease from post-infection day three; two of the three sows died suddenly on post-infection day five, while the third was euthanized on the same day for animal welfare reasons. Following necropsy, the presence of ASFV DNA was detected in tonsils, spleen and lymph nodes of some of the fetuses, but the levels of viral DNA were much lower than in these tissues from the sows. Thus, only limited transplacental transmission occurred during the course of this experiment. These studies contribute towards further understanding about the spread of this important viral disease in domestic pigs.

## 1. Introduction

African swine fever virus (ASFV) is the cause of a serious hemorrhagic disease of domestic pigs and other members of the family *Suidae*, including wild boar [1,2]. The infection can lead to high case fatality rates in domestic pigs and in wild boar. The causative agent is classified within the genus *Asfivirus*; it is a large DNA virus belonging to the *Asfarviridae* family, indeed it is the only member of this family [3]. The virus was first identified in Africa [4] and numerous (at least 24) distinct genotypes of the virus exist; these are classified based on the sequence of the VP72 gene [5,6,7,8,9]. The size of the viral genome varies between strains and is about 170–190 kbp in length. Approaching 200 genes are closely spaced along the genome and are transcribed from the two different strands of the genome [10,11,12,13]. The functions of many of these genes are currently unknown.

On occasions, the virus has spread outside of Africa, e.g., in the late 20th century to the Iberian Peninsula and Italy (Sardinia) in Europe, but also to South America and Cuba [1]. Furthermore, in 2007, a genotype II strain of the virus was introduced into Georgia and has since spread through the Caucasus to Russia, and in 2014 it entered the European Union (EU). Wild and domestic pigs in multiple European countries have been infected; the affected countries include the Baltic countries, Poland, Czech Republic (now declared free again of ASFV), Romania, Bulgaria, Hungary, Belgium (also now declared free of ASFV), Germany (since 2020) and mainland Italy (since 2022). In 2018, the virus also spread into Asia and has caused massive problems for the pig production industry in China and nearby countries, e.g., Vietnam, Laos, Cambodia and South Korea [14].

Different strains of the virus vary in their virulence. The genotype II virus, which currently causes most of the outbreaks outside Africa, is associated with very high levels of mortality (close to 100%) but some more attenuated variants of this virus have arisen, e.g., in parts of Europe [15,16] and in China [17]. Currently, there are neither commercially available vaccines nor anti-viral agents to control the disease. However, attempts to produce live-attenuated vaccines are in progress; these have mainly focused on deleting specific genes from ASFV to reduce its virulence in swine without preventing efficient growth in cell culture [18,19,20]. 

It has been established that, in Africa, ASFV infects both soft ticks (genus *Ornithodoros*) and warthogs, without causing apparent disease [21,22]. A sylvatic cycle exists with virus replication in both of these hosts. ASFV is unique in being the only known DNA arbovirus [23]. Outside Africa, transmission of the virus is believed to occur mainly by direct or indirect contact between infected wild or domestic pigs, without the involvement of soft ticks, although some aspects of virus transmission have been poorly characterized [24]. 

The disease is characterized by high fever (often >41 °C) together with a range of clinical signs including lethargy, anorexia, skin hemorrhages and bloody diarrhea, which can occur within a few days of infection. Death can occur with few external signs, but postmortem examinations often indicate enlargement of the spleen and lymph nodes, as well as internal bleeding. The virus replicates in macrophages and monocytes within the blood and is also present at high levels within the lymph nodes, tonsils and spleens of infected animals [2,25,26]. Transmission of the virus between pigs readily occurs by direct contact between animals, and also indirectly by contact with virus-infected material (e.g., blood) [24]. Furthermore, airborne infection over short distances (within enclosed buildings) has been described [27,28], and infection of pigs has also been observed following bites from or ingestion of insects (stable flies, *Stomoxys calcitrans*) that had fed on ASFV-containing blood, in the absence of virus replication within these insects [29].

In order to gain knowledge of the properties of the genotype II ASFV, which has spread widely across Europe and Asia, and to analyze its transmission, experimental studies were performed under defined conditions, in both young healthy pigs and in pregnant sows. The main objective of the experiment with young pigs was to study the course of disease in Danish pigs of different sanitary status. A key objective of the experiment with sows was to determine whether ASFV was transmitted between the sows and the fetuses. If so, it was important to determine which fetal tissues were susceptible to infection and could provide a marker for transplacental transmission of the virus. 

Preliminary reports of these studies have been published (in Danish) previously [30,31].

## 2. Materials and Methods

### 2.1. Animal Experiments

Two experimental studies were conducted on animals within the BSL3 high biocontainment facility at Lindholm. All animals were allowed one week of acclimatization after transportation into the biocontainment facility before the start of the experiment. The animals were provided with a commercial diet once a day and water was provided *ad libitum*. Straw was used for bedding.

The genotype II ASFV used in these experiments was initially obtained from Georgia in 2007 and hence is termed Georgia/2007. A sample of spleen from a pig infected with this virus was kindly provided by the EURL-ASF, Madrid, Spain. For the experimental infections of young pigs (experiment 1), spleen material was diluted 1:40 in 0.85% NaCl (for intranasal inoculation) or 1:20 in 0.85% NaCl (for intramuscular inoculation). For the inoculation of sows (experiment 2), the virus was isolated from spleen material, essentially as described previously [28] in porcine pulmonary alveolar macrophages (PPAM). Briefly, for isolation of the virus, a suspension of PPAM cells (10 mls with 2 × 10^6^ cells/mL in medium with 5% FCS) was inoculated with a clarified 25% spleen suspension. After three days of incubation at 37 °C, (with 5% CO_2_), the first passage virus was harvested by freezing and thawing prior to the inoculation of the animals.

The titers of the virus samples used for inoculation of the pigs in each experiment were determined by end-point titration in PPAM. Following three days of incubation (as above), virus-infected cells were identified following fixation and staining of the cells using an immunoperoxidase monolayer assay (IPMA) essentially as described previously [32]. Briefly, the infected cells were stained using ASFV antibody-positive swine serum followed by horseradish peroxidase conjugated anti-swine IgG (Sigma-Aldrich, St. Louise, MO, USA) plus hydrogen peroxide and scored using a light microscope. The virus titer (as TCID_50_/mL) was calculated as described previously [33].

Experiment 1 included 17 weaner pigs, 8 weeks of age, that were separated into two groups on the basis of different health status criteria. Group 1 (9 pigs) had a uniquely high sanitary status originating from the National Veterinary Institute’s own herd at Lindholm (as described previously [28,34]). Group 2 (8 pigs) were specific pathogen free (SPF) high health status animals (SPF system based on Danish standards), as described previously [34]. All pigs were judged as healthy by visual inspection and rectal temperatures were within a normal range on the day of inoculation. In both groups, four pigs were inoculated with ASFV Georgia/2007 on post infection day (PID) 0. For two of these pigs in each group (nos. 1, 2 plus 10 and 11), intramuscular (i.m.) injection (using spleen homogenate (5%) in 0.85% NaCl (with 5.9 log_10_ TCID_50_ at back titration) was used, and for the other two pigs in each group (nos. 3, 4, 12 and 13) intranasal (i.n.) inoculation (using spleen homogenate (2.5%) in 0.85% NaCl (with 5.9 log_10_ TCID_50_ at back titration) was performed. The remaining pigs in each group were housed in the same pen as the inoculated animals and are termed “in contact” pigs. The two groups were housed separately in isolated units, thus with no physical contact and no air contact between them. 

For experiment 2, three pregnant sows from a commercial herd, each at about 100 days of gestation, were inoculated intramuscularly with ASFV Georgia/2007 (using a dose of 4.3 log_10_ TCID_50_ /sow) on PID 0. The 3 sows were housed individually in pens within the same isolation unit. 

In each experiment, clinical assessments were made on a daily basis and the pigs were individually assigned a clinical score (CS) each day, essentially as described previously [28]. Briefly, the CS was based on the following parameters: rectal temperature, appetite, alertness and recumbency, body condition, presence of skin hemorrhages, joint swelling, respiration, ocular discharge, abnormalities observed in gastrointestinal and urinary tracts, and neurology. Each parameter was assigned a score between 0 and 6, where 0 = normal and a positive score = deviation from normal. The maximum score was 40. When pre-determined humane end points were reached, the pigs were euthanized through intravascular injection of a lethal dose of Pentobarbital. 

In experiment 1, blood samples were collected from each surviving pig on PID 1, 3, 6 and 9, and at euthanasia. In experiment 2, blood, nasal swabs and fecal samples were planned for collection on PID 0, 4, 7, 11 and 14 (but the experiment was terminated early, see below). Gross pathological examinations were performed at necropsy, and placentas together with selected tissues (tonsil, lymph nodes and spleen) from the sows and fetuses were collected for further examination. Sera from blood samples were tested for antibodies against the ASFV VP72 protein using the Ingezim PPA Compac ELISA (^®^INGENASA-INGEZIM PPA COMPAC K3 INGENASA) according to the manufacturer’s instructions.

### 2.2. Pre-Processing of Samples

Organ homogenate suspensions (25%, *w*/*v*), vomit and fecal homogenate suspensions (10%, *w*/*v*) were prepared in Eagles minimum essential medium (Sigma-Aldrich), as described previously [35]. Following centrifugation, the clarified supernatants were stored at −80 °C until further analysis.

### 2.3. ASFV DNA Detection by Quantitative Real-Time Polymerase Chain Reaction (qPCR)

From blood samples obtained in experiment 1, viral DNA was extracted using a combination of acid phenol extractions and binding nucleic acid to silica particles (as described by [36]). Extracted DNA samples were tested for the presence of ASFV DNA by quantitative (q) PCR using the Mx3005P qPCR system (Agilent Technologies, Santa Clara, CA, USA), essentially as described by Braae et al. [37].

For experiment 2, viral DNA was extracted from samples of sera, together with nasal swabs and supernatants of homogenized fecal, vomit and tissue samples using a MagNA Pure LC system (Roche, Basel, Switzerland) with the MagNA Pure LC Total Nucleic Acid Isolation Kit (Roche) and the Total NA External Lysis protocol. Viral DNA was extracted from tissue samples obtained from the sows and fetuses using the procedure described previously [36]. Extracted DNA samples were tested for the presence of ASFV DNA by quantitative (q) PCR using the Mx3005P qPCR system (Agilent Technologies), as described [38]. 

## 3. Results

### 3.1. Course of Infection in Young Pigs (Experiment 1)

**Figure 1:**
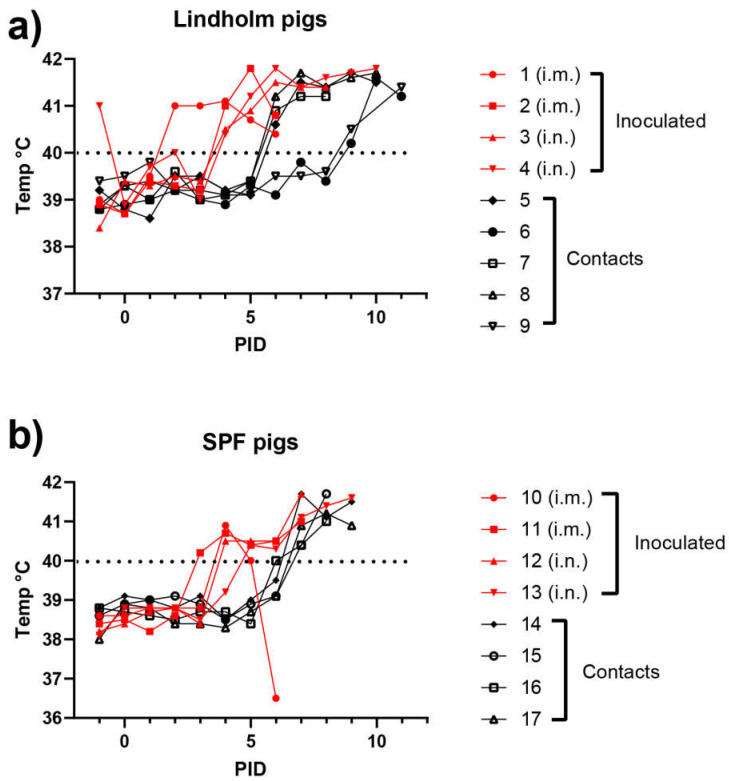
Rectal temperatures of ASFV-infected pigs using animals obtained from Lindholm’s herd (**a**) or from a commercial supplier of SPF pigs (**b**).

In experiment 1, two groups of young pigs were used; one group (numbered 1–9) comprised animals from Lindholm’s own herd with a very high sanitary status, while the second group (numbered 10–17) were conventional Danish SPF pigs. In each group, two pigs were inoculated intramuscularly and two pigs were inoculated using the intranasal route. Among the Lindholm pigs (Group 1), signs of infection were detected as early as PID 2, as judged by an elevated temperature (>40 °C). By PID 4, all four inoculated pigs in this group were showing symptoms of disease (Figure 1a). Similarly, the 4 inoculated SPF pigs all displayed fever by PID 4, and indeed one had shown fever as early as PID 3 (Figure 1b). The i.m. route of infection appeared to result in a slightly earlier onset of fever in both groups of animals. In Group 1, three of the contact pigs also developed fever by PID 6, but two others only displayed symptoms from PID 9 (Figure 1a). 

**Figure 2:**
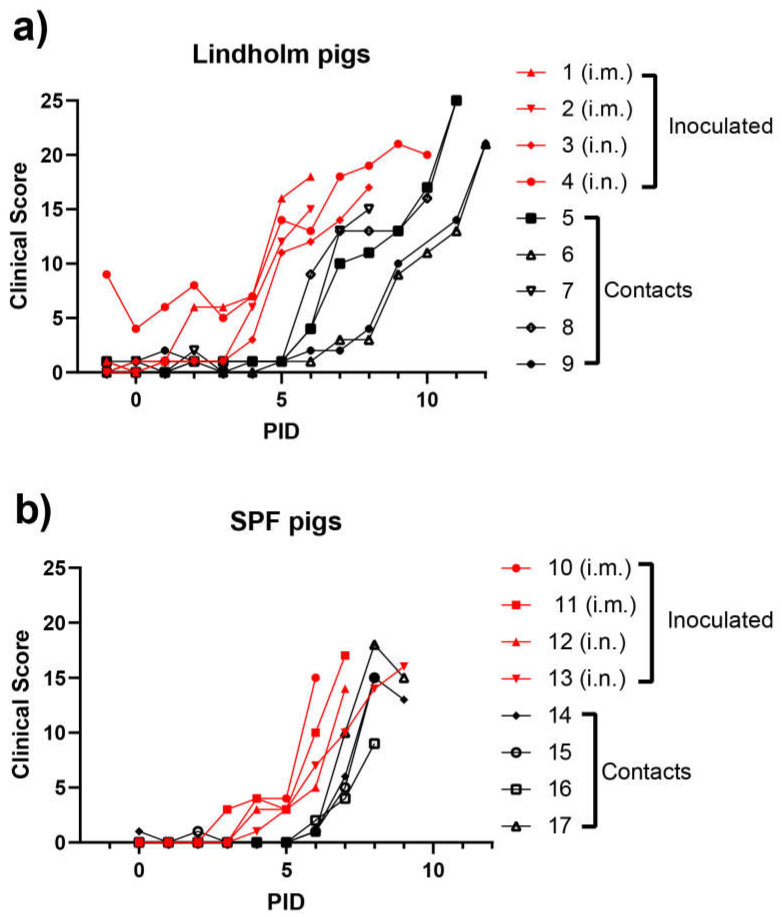
Clinical scores from ASFV-infected pigs using animals obtained from Lindholm’s herd (**a**) or from a commercial supplier of SPF pigs (**b**).

The contact pigs in Group 2 all displayed fever by PID 7, i.e., infection spread to the contact pigs with a delay of only about three days (Figure 1b). From PID 2 and PID 3, consistent with the changes in rectal temperatures, clinical signs typical of ASFV infections were observed in the inoculated animals, in the form of recumbency, inappetence, dyspnea, bleeding from the rectum, vomiting and diarrhea, with increasing severity over time. Seizures were recorded for one individual (pig 3). As a consequence, the CS for the pigs progressively increased (see Figure 2a,b). As with the earlier appearance of fever, the i.m. route of infection appeared to result in a slightly earlier onset of clinical disease. The contact animals all showed a similar pattern of disease with about four days of delay. Thus, under the conditions of this experiment, contact transmission was very efficient.

Young pigs (approximately 8 weeks of age) from Lindholm’s own herd (panel a) or from a SPF pig supplier (panel b) were inoculated (i.m. or i.n., as indicated) with ASFV Georgia/2007 on PID = 0, and kept with “in contact” animals for the duration of the experiment. Rectal temperatures were recorded on a daily basis. The dotted line highlights the temperature of 40 °C.

At PID 9 (SPF pigs) or PID 12 (Lindholm pigs), the experimental studies were terminated. At PID 6, the first pigs (i.m. inoculated) died. In the following days, in total, five pigs died and the remaining 12 pigs were euthanized due to animal welfare reasons.

#### 3.1.1. Pathological Findings (Experiment 1)

Following death or euthanasia (for animal welfare reasons) at between PID 6 and 12, each of the animals was examined at necropsy. Some of the pigs showed very few pathological lesions, but the vast majority showed bleeding in the abdomen, with haemorrhagic enlarged lymph nodes. In many of the animals, internal petechial bleeding was also observed along with atrophy of the tonsils and thymus. The pathological lesions did not differ markedly between the two groups of pigs.

In each group of either Lindholm pigs (panel a) or SPF pigs (panel b), four pigs were inoculated (i.m. or i.n., as indicated) with ASFV Georgia/2007 and the remaining animals were kept in contact with the inoculated animals throughout the rest of the experiment. Clinical scores were assessed for each animal on a daily basis (as described in Section 2).

#### 3.1.2. Virological Findings (Experiment 1)

**Figure 3:**
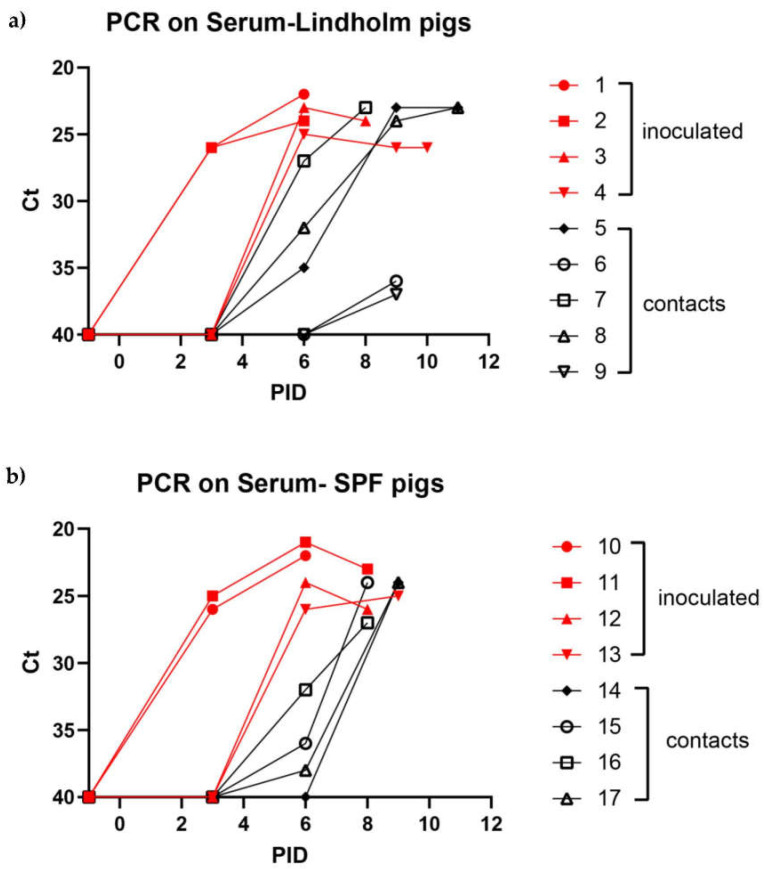
Detection of ASFV DNA in pig sera collected from animals obtained from Lindholm’s herd (**a**) or from a commercial supplier of SPF pigs (**b**).

The presence of ASFV in serum samples, collected (normally at three-day intervals) throughout the course of the infection, was determined using qPCR. ASFV DNA was readily detected (Ct < 30) in the sera of inoculated pigs from PID 3, and appeared in the sera of contact pigs from PID 6 (Figure 3a,b). By PID 9, all of the pigs had become infected and ASFV was present in their blood. A similar profile of infection and transmission was seen in the SPF pigs and the Lindholm pigs (Figure 3a,b) but the i.m. inoculated animals in each group (nos. 1, 2, 10 and 11) had virus in their serum earlier than the i.n. inoculated pigs. Sera collected from each of the pigs on the day of their euthanasia were also tested by ELISA for the presence of anti-VP72 antibodies, pigs 2,3,4,6,7,9,10,11,14 and 15 were all scored negative in this test, whereas pigs 1,5,8,12 and 13 gave inconclusive/borderline results at this time (6–11 days post inoculation).

In each group of either Lindholm pigs (panel a) or SPF pigs (panel b), four pigs were inoculated (i.m. or i.n., as indicated) with ASFV Georgia/2007, and the remaining animals were kept in contact with the inoculated animals throughout the rest of the experiment. ASFV DNA was extracted from pig serum samples and assayed by qPCR. If no Ct value was obtained after 40 cycles, the sample was given a value of 40. Values are presented for the samples from inoculated and “in contact” animals collected on pre-selected days. Note: solid lines have been used to link the data points for the purpose of clarity but should not be used to infer levels of viral DNA present in serum at intermediate time points.

### 3.2. Course of Infection in Pregnant Sows (Experiment 2)

**Figure 4:**
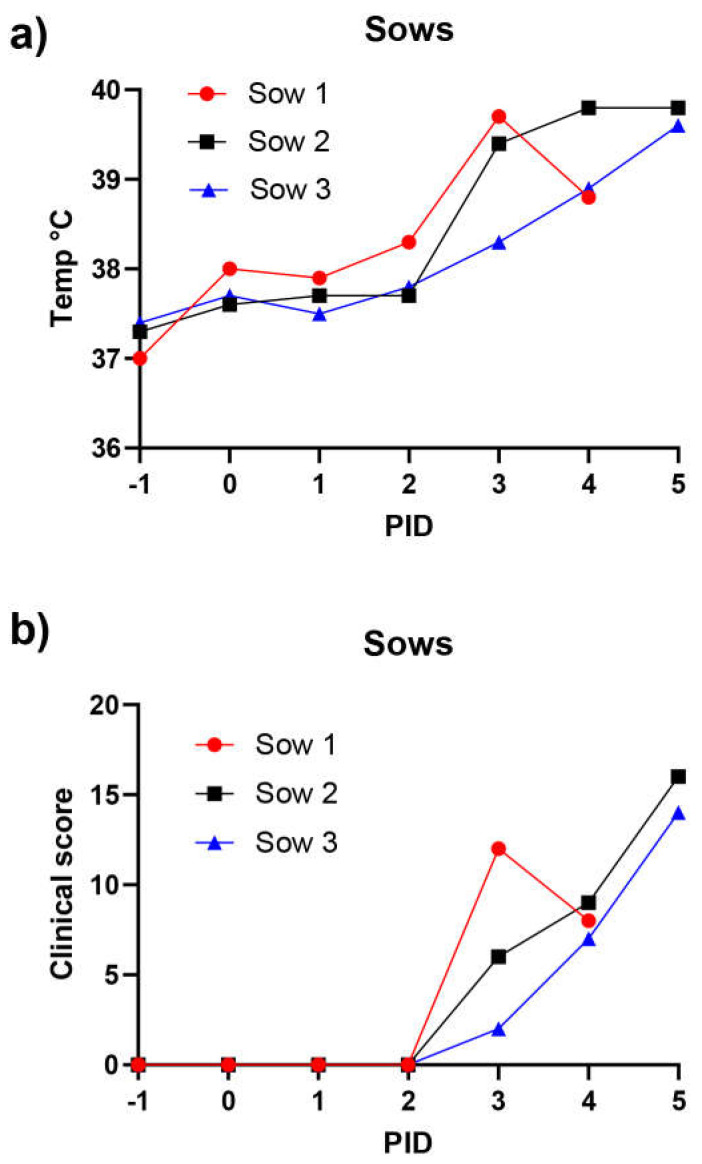
Rectal temperatures (**a**) and clinical scores (**b**) in ASFV-inoculated sows.

In experiment 2, three pregnant sows were inoculated by intramuscular injection of ASFV Georgia/2007 (4.3 log_10_ TCID_50_/sow) at PID 0. Prior to the inoculations, each of these sows was found clinically healthy by inspection, and rectal temperatures between 37 and 38 °C were recorded. During the two days following the inoculation, the sows remained clinically unaffected and had temperatures within this normal range. However, from PID 3, rectal temperatures increased to between 38.3–39.7 °C (Figure 4a). The temperature of sow 1 declined on PID 4 and this sow unexpectedly died before clinical assessment on PID 5. The temperatures of sows 2 and 3 were still markedly elevated (nearly 40 °C, Figure 4a). 

The CS values assessed throughout the course of the experiment are shown in Figure 4b. Unspecific clinical signs were evident from PID 3, including lethargy and markedly reduced appetite. The course of the disease was short; lethargy and inappetence continued on PID 4 and were then followed by abrupt completion of the experiment on PID 5, when sow 1 was found dead during the morning inspection. Sow 2 also died, despite close monitoring, later on the same day following convulsions and vomiting. Hence, due to animal welfare considerations the remaining sow (sow 3) was euthanized on the same day. Sow 3 was markedly lethargic at euthanasia. 

#### 3.2.1. Pathological Findings (Experiment 2)

At necropsy, tissue samples were collected from the three sows and also from ca. 20 fetuses per sow. During postmortem examination of the three sows, lesions were only found in sow 1, which died unexpectedly at PID 4. No lesions were observed in sow 2, which died following convulsions, nor in sow 3, which was euthanized due to animal welfare considerations. During necropsy of sow 1, bloody discharge was observed around the vulva, perhaps indicating that the sow had been close to labor. Pathological findings included hemorrhagic lymph nodes and mildly red-colored intestines. In some of the fetuses from sows 1 and 2, lesions included hemorrhagic tonsils, discoloring of the edge of the spleen and an increased amount of fluid in the abdomen. No lesions were observed in the fetuses from sow 3.

#### 3.2.2. Virological Findings (Experiment 2)

Samples of serum, together with nasal swabs and feces, were collected throughout the course of the experiment and assayed by qPCR for the presence of ASFV DNA. High levels of viral DNA (Ct < 22.5) were present in serum by PID 4 (and were maintained on PID 5 in the surviving sows), but as expected, no ASFV DNA was detected in the serum prior to inoculation (Table 1). 

One of the sows (sow 2) vomited on PID 5, shortly before the termination of the experiment. The vomit contained low levels of ASFV DNA (Ct = 37.0). 

Following necropsy (on PID 5), it was found that the infected sows had high levels of ASFV DNA in tonsils, mesenteric and ventricular lymph nodes and spleens (Ct < 20 in each case) (see Table 1). In contrast, only low levels of viral DNA were detectable in the corresponding tissues from the three fetuses from each sow that were analyzed in detail (Table 1). The highest level of viral DNA detected in the fetal samples was a Ct = 34.2 (in spleen), and many of the samples were completely negative (Ct > 40) (see Table 1). The tonsil samples were tested from all of the fetuses. In total, between two and six of the 20–22 fetuses obtained from each s, had low levels of viral DNA (Ct > 34) in these tissues, in contrast with the high levels of virus (Ct < 20) within these tissues from the infected sows, (i.e., over 1000× higher levels of ASFV DNA were present in the adult tissues). Thus, it appears that the transfer of ASFV from the sows to the fetuses was very limited during the course of the experiment (up to PID 5). 

Three pregnant sows (at about 100 days of gestation) were inoculated with ASFV Georgia/2007 on PID = 0. The rectal temperatures (panel a) and clinical scores (panel b) for the pigs were assessed on a daily basis (from PID= −1). One sow died before morning inspection on PID = 5, a second died unexpectedly on PID 5, and the third was euthanized on the same day for animal welfare reasons.

No antibodies to ASFV were detected in serum samples from the three sows taken on days PID 0, PID 4 and PID 5 (data not shown). 

## 4. Discussion

Infection with ASFV Georgia/2007 was produced by intramuscular or intranasal inoculation of typical, healthy (SPF) Danish pigs (approximately 8 weeks of age) and healthy pigs from Lindholm’s own herd with very high sanitary status (monitored and known to be free of a wide range of pig pathogens). The first clinical signs of infection (elevated temperatures) became apparent from as early as PID 2 and were observed in each of these animals by PID 4. Spread to “in contact” pigs within the same pen occurred efficiently (using equal numbers of inoculated and “in contact” pigs) and there was a delay of 3–4 days between the appearance of infection in the inoculated animals and in the “in contact” animals. The profiles for the two groups of young pigs indicating temperatures, clinical pathological findings and virological findings were very similar, suggesting that the infection in healthy pigs developed independently of the sanitary status of the pigs.

It should be noted some of the pigs died without showing ASFV-specific clinical and pathological lesions. This has been described previously [39]; rather general symptoms are often observed, e.g., mild anorexia, lethargy and recumbency, while more specific signs of ASF such as haemorrhages and bloody diarrhoea are only seen in some animals. Thus the spectrum of clinical observations, from little apparent disease to sudden death, should be borne in mind for the control of the disease.

The results obtained here in experiment 1 were broadly consistent with other studies using the genotype II ASF virus [28,39,40]. Thus, these data strengthen confidence in the parameters used for modelling potential outbreaks of ASFV in domestic pigs. It was interesting to note that among the Lindholm pigs the transmission of the infection from the inoculated animals to the contact animals (see Figure 1a, Figure 2a and Figure 3a) appeared to occur in two steps. Three of the pigs (numbers 5, 7 and 8) seemed to become infected directly from the inoculated pigs and showed a delay of about three days in the appearance of clinical signs (Figure 2a). However, the infection of two pigs (numbered 6 and 9) was further delayed by at least two days (Figure 1a, Figure 2a and Figure 3a). It could be that this represents variation in sensitivity to infection between animals, but it might indicate that these final infections only resulted from transmission of the initial infections in the contact animals (numbered 5, 7 and 8). This may suggest a limit to the period of time during which infected animals are able to transmit the virus to susceptible hosts, and is worthy of further investigation. This would also appear to be consistent with the very limited ability to observe virus transmission from an environment highly contaminated with the virus, following the removal of ASFV-infected animals (see [35]). 

We are unaware of other studies assessing the outcome of the genotype II ASFV Georgia/2007 during late stages of pregnancy. Earlier studies had shown abortions induced by a low-virulence strain of ASFV and found that fetal placenta, amniotic fluid and fetal blood all contained detectable ASFV, but in general most fetal tissues had little or no detectable virus, as determined using immunostaining methods [41]. Using PCR, Antiabong et al. [42] detected ASFV DNA in certain fetal tissues (liver, placenta and lung) but not others (kidney and heart), following infection of a single sow, suggesting that transplacental transmission of the virus can occur. From the studies performed here, it is clear that the pregnant sows were highly susceptible to infection by ASFV Georgia/2007 and developed typical clinical signs of the disease, including fever along with inappetence and recumbency. High levels of the virus were present in the tonsils, lymph nodes and spleens of the infected sows, but very limited transplacental transmission of the virus to the fetuses could be demonstrated. Only low-level signals (Ct > 34) were detected in the tonsils, lymph nodes and spleens of a few of the fetuses (this was at least a 1000-fold lower level of viral DNA than in the corresponding tissues from the sows). This may simply be a matter of the speed at which the infection occurred in the sows (two of the three sows died unexpectedly on PID 5 and the third was euthanized for animal welfare reasons on the same day). However, if very efficient transfer of the virus across the placenta from the blood of the sow occurred early in infection, then it may be expected that significant levels of the virus would have been detected in the fetuses. Clearly, the number of sows infected here was necessarily small. Thus, in other environments, some sows could potentially survive ASFV infection, and hence some live offspring could potentially be produced from an infected and recovered sow. The spread of virus to the tissues of some of the fetuses suggests the infection could already be established and may then circulate within the newborn piglets.

## 5. Conclusions

The genotype II strain of ASFV, originally isolated from Georgia in 2007, was highly pathogenic in both young pigs and in adult sows. Efficient transmission of the virus from infected animals to “in contact” animals was observed, with about a 3–4-day delay in the time course of infection. However, very limited transmission of ASFV from the pregnant sows to fetuses occurred during the five-day period between inoculation and death of the sows. High levels of viral DNA were detectable in the serum, tonsils, spleen and lymph nodes of the sows, but only low levels of the virus genome were detected in the tonsils of some of the fetuses and most were virus negative. This suggests that transplacental transmission of ASFV occurs, but is inefficient.

## Figures and Tables

**Table 1:** Presence of ASFV DNA in samples from ASFV-infected sows and three of their fetuses collected during necropsy.

	Sow 1	Fetus 1-1	Fetus 1-2	Fetus 1-3	Sow 2	Fetus 2-1	Fetus 2-2	Fetus 2-4	Sow 3	Fetus 3-1	Fetus 3-2	Fetus 3-6
Sample												
Ser. PID 0	>40				>40				>40			
Ser. PID 4	20.7				22.3				21.3			
Ser. PID 5	-				22.3				20.4			
Tonsil	18.3	37.5	>40	>40	17.7	36.9	>40	36.3	18.1	36.5	37.5	35.4
Spleen	18.8	35.8	>40	>40	18.5	>40	35.9	>40	17.2	>40	34.3	34.2
Ln Mes	19.1	>40	>40	>40	17.7	36.5	34.5	>40	18.1	>40	36.0	35.0
Ln Ven	17.4			>40	18.2			36.5		36.1		37.6
Placenta	31.2								29.1			
Nasal sb	29.8				>40				34.0			

The ASFV DNA was detected by qPCR as described in Materials and Methods. Abbreviations: Ser. = serum; Ln Mes = mesenteric lymph node; Ln Ven= ventricular lymph node; Nasal sb = nasal swab. Serum samples were collected from the sows prior to inoculation on PID 0, then on PID 4 and on PID 5 (when possible). All tissue samples were collected during necropsy. Numbers shown are Ct values from qPCR assays, colour coded as follows: <20 in yellow; 20–30 in green; 30–40 in blue and >40 in grey. Note: lower Ct values correspond to higher levels of ASFV DNA.

## Data Availability

All necessary data are contained within the article.
